# Therapies and Challenges in the Post-Stroke Aphasia Rehabilitation Arena: Current and Future Prospects

**DOI:** 10.3390/medicina59091674

**Published:** 2023-09-17

**Authors:** Anastasios M. Georgiou, Maria Kambanaros

**Affiliations:** The Brain and Neurorehabilitation Lab, Department of Rehabilitation Sciences, Cyprus University of Technology, 3041 Limassol, Cyprus; maria.kambanaros@cut.ac.cy

**Keywords:** aphasia, research, recruitment

## Abstract

Aphasia is a serious consequence of stroke that results in a breakdown in communication. The course of aphasia recovery differs between afflicted individuals, and responsiveness to treatment cannot be predicted. Aphasiologists continue to investigate numerous behavioral treatment protocols that have shifted their focus to complimentary rehabilitation strategies. The aim of this study is threefold. First, to summarize the different categories of aphasia interventions post-stroke, considering their respective protocols, and present available evidence on the effectiveness of those protocols. Second, to document the challenges regarding the prediction of aphasia treatment response post-stroke in individual patients. Third, to report the challenges faced by researchers in recruiting people with aphasia (PWA) for treatment studies, and provide recommendations on how to increase participant recruitment and retention. This study provides up-to-date information on (i) effective therapies and aphasia recovery processes, and (ii) research recruitment hurdles together with potential strategies for overcoming them.

## 1. Introduction

Aphasia is an acquired language disorder (derived from the Greek word “afaˈsia”) that affects spoken and/or written language resulting from brain injury (e.g., stroke). Apart from having devastating consequences on communication, aphasia also undermines the individual’s sense of identity with serious effects on family life and social participation. Loss of independence, limitations in activities of daily living, decreases in social networks [1] (poor quality of life (QoL) [2], and long-term disability [3] are some of the major repercussions of aphasia. 

The etymology of the word aphasia implies that people that experience it are characterized by a complete absence of language; that is, they have global aphasia. Nevertheless, in clinical practice, the global aphasia phenotype is uncommon and even though people that exhibit it often show late recovery [4], they more typically evolve to have Broca’s aphasia [5]. Thus, although the term “dysphasia” would sound more appropriate for all aphasia syndromes except global aphasia, in speech and language therapy (SALT), the convention is to use the term “aphasia” in all cases of acquired language disorders resulting from brain injury, irrespective of their type, severity, and stage of characterization. In brief, aphasias are classified into non-fluent (global, mixed transcortical, Broca’s, transcortical motor, and aphemia/pure word mutism) and fluent (Wernicke’s, transcortical sensory, conduction, and anomic) [6]. Non-fluent aphasias share a common speech deficit, that is, non-fluent speech. Fluent aphasias reflect (almost) intact verbal fluency.

This study has three aims. First, to summarize the different categories of post-stroke aphasia interventions, provide an overview of their respective protocols, and provide available evidence on the effectiveness of the interventions. The goal is to deepen the reader’s understanding of the aphasia rehabilitation arena. A suite of treatment approaches has been implemented over time, ranging from impairment-based, compensatory, psychosocial, and pharmacological, to list a few. Clinical speech and language pathologists need to become familiar with the relevant treatment studies to engage in evidence-based aphasia therapies to improve the lives of people living with aphasia (PWA). As accurate prediction of treatment response in PWA is important (who benefits/who does not), the second aim of this study is to highlight the challenges related to the prediction of individualized aphasia treatment response. The third aim of this study is to document the challenges faced by researchers in recruiting participants for post-stroke aphasia rehabilitation studies. Further, some recommendations on how to increase participant recruitment and retention are proposed. This research provides up-to-date information on (i) effective therapies and aphasia recovery processes, and (ii) research recruitment hurdles together with potential strategies for overcoming them.

## 2. Post-Stroke Aphasia Therapies 

Globally, there are over 13.7 million new strokes each year [7]. Aphasia, being a significant consequence of stroke, affects more than a third of all stroke survivors [8]. Considering those figures on an annual basis and global scale, the need for effective aphasia treatments is imperative. 

Four categories of aphasia interventions, either in isolation or combined, have been explored over the years: (1) pharmacological therapies (i.e., growth factors, monoclonal antibodies, cell-based therapies, and drugs); (2) behavioral (SALT); (3) additional therapeutic approaches (e.g., technological aids); and (4) non-invasive brain stimulation-based therapies (NIBST) (i.e., Transcranial Magnetic Stimulation (TMS) and Transcranial Direct Current Stimulation (tDCS)).

### 2.1. Pharmacological Therapies 

Several promising pharmacological agents have been explored either in isolation or together with other forms of treatments (e.g., SALT) to promote improvements in aphasia symptoms. Drugs that show moderately positive outcomes include acetylcholinesterase inhibitors and dextroamphetamine sulfate [9], while selective serotonin reuptake inhibitors may also help aphasia recovery [10]. Piracetam, a well-explored agent, is a nootropic drug that enhances cognitive functions by facilitating vascular microcirculation. In a systematic review and meta-analysis, it was found that piracetam enhances written language skills but plays a limited role in the recovery of overall language deficits, and its short-lived benefits represent a major drawback of this agent [11]. On the other hand, memantine—a N-methyl-D-aspartate receptor antagonist—has shown potential for sustained language improvement in chronic post-stroke aphasia especially when it is combined with Constraint-Induced Aphasia Therapy (CIAT) [12]. The main limitation of most studies is that many are open label or cross-sectional trials with small numbers of participants. The lack of sham (control) conditions leaves the trials open to likely placebo effects (i.e., believing that a procedure/treatment will work leading to symptom modulation by the brain) and makes it impossible to explore whether the observed language gains are due to (i) placebo effects or (ii) pharmacotherapy alone or (iii) a combination of drugs and other forms of treatment. 

In sum, the effect sizes of most trialed pharmacological treatments for aphasia are small and the functional and structural correlates of the possible positive effects are mostly unknown [13]. There is one notable exception though. Berthier et al. [14] conducted a randomized controlled trial with 26 participants and found that the administration of 10 mg of donepezil (a cholinesterase inhibitor) in combination with two hours of SALT per week improved picture naming (Cohen’s d = 0.92) and the severity of aphasia (Cohen’s d = 0.87). Such promising findings highlight the potential synergistic effect of donepezil and SALT on language gains in chronic post-stroke aphasia. Nonetheless, this was a study with a small sample size and therefore further research is needed to confirm such findings, and to fully understand the potential benefits and limitations of using donepezil in aphasia rehabilitation.

Unfortunately, there is also evidence supporting the detrimental effects of several drugs on post-stroke recovery (e.g., a-blockers [15]; dopamine antagonists [16]; carbamates [17]). Overall, currently no pharmacological interventions for aphasia are approved by the United States Food and Drug Administration (FDA) [18], and, according to a large-scale study, behavioral language activities strongly remain the gold standard for aphasia treatment [19].

### 2.2. Behavioral Therapies 

SALT protocols are continuously being explored, and, as a result, several different types of aphasia therapy approaches have been implemented over the years. Following the International Classification System (ICF) of the World Health Organization (WHO) [20], several aphasia interventions focus on one or more of the following: speech and language deficits, personal and activity limitations, and environmental barriers. Aphasia interventions can be also divided into those targeting speech and language deficits (i.e., didactic, behavioral modification, stimulating) and those emphasizing functional communication rather than language recovery (i.e., pragmatics school) [21]. Findings from two systematic reviews indicate that therapies that target language impairment are effective [22,23]. Even though for several approaches there is robust evidence supporting their efficacy (while for others the evidence is emerging), there are limitations concerning the maintenance and generalization of gains beyond trained items for at least some PWA. Also, the expectation of transfer of skills from one domain to another (e.g., from speaking to reading) is currently unexplored territory. 

Up until now, there has been limited understanding around the standardization of aphasia therapy protocols, resulting in a vast collection of behavioral treatments for PWA. For example, in the systematic review of Husak et al. [24] in which the effects of SALT initiated within four months of stroke-induced aphasia onset were explored, it was reported that there is still a need for high-quality research to define which types of therapy are most effective during this period. Despite notable progress, as evidenced by the publication of five clinically meaningful studies [19,25,26,27,28], each with a substantial sample size and rigorous methodology, disparities in findings persist among these studies, highlighting the ongoing need for further research to achieve the desired standardization of aphasia treatments. The COMPARE trial by Rose and colleagues [25] found that constraint-induced or multimodality aphasia treatment is better than usual care in terms of language gains for chronic aphasia post-stroke. The Big CACTUS trial [26] demonstrated the superiority of self-managed, computerized SALT over usual care or attention control for chronic aphasia post-stroke. Breitenstein et al. [19] reported that 3 weeks of intensive SALT resulted in significant improvements in verbal communication for people with chronic aphasia post-stroke. The VERSE study by Godecke and colleagues [27] revealed that early, intensive aphasia therapy did not improve communication recovery within 12 weeks post-stroke compared to usual care. Also, in their exploration of early cognitive–linguistic treatment for post-stroke aphasia, the researchers of the Rotterdam Aphasia Therapy Study-3 [28] demonstrated results that do not show a clinically relevant effect of very early cognitive–linguistic treatment on everyday language.

An overview of important and commonly used aphasia treatment approaches is presented below. The list that follows is not comprehensive, and it should be noted that in the different phases of aphasia (acute versus chronic), a different therapeutic approach may be suitable due to the different neurophysiological mechanisms underpinning each phase. 

#### 2.2.1. Phonomotor Treatment 

Phonomotor Treatment (PMT) aims to boost word retrieval through the training of phonological skills based on the assumption that phonology is fundamental for all language functions [29,30]. It is believed that, as in any language, the number of phonemes and their respective sequences are limited, and by cultivating phonological skills in PWA, gains in trained and untrained words are feasible [31]. For phonological training purposes, a multimodal approach is adopted (i.e., mirrors, mouth pictures, written representations, kinesthetic feedback, etc.). Training starts with single phonemes and gradually progresses to phoneme sequences in nonwords and real words. Crucially, Socratic maieutics, a form of argumentative dialogue between people, is used to urge PWA to reflect on their own attempts. Recent evidence shows that PMT has the potential to enhance the confrontation naming of trained items with some generalization to and maintenance of untrained words [32]. PMT has also the potential to lead to improvements in discourse production [33], revealing a transfer effect from phonology to discourse. 

#### 2.2.2. Semantic Feature Analysis 

Semantic Feature Analysis (SFA) also facilitates word retrieval and relies on theoretical models suggesting that the human semantic system is organized into concept networks [34]. When a person attempts to retrieve a word, all characteristics of the target concept together with features for other associated concepts are activated. The feature(s) that are activated the most are selected first and then the respective phonological representation(s) and motor program execution(s) are activated. PWA may activate the wrong concepts, and, as a result, they articulate incorrect, but semantically related, words in place of the target word. During treatment, PWA are prompted to use feature analysis charts that provide information regarding use, location, physical properties, and association concepts to generate semantic features of target concepts. Successful attempts strengthen semantic networks and help with lexical retrieval. Several studies suggest that SFA leads to word-finding improvements in trained items e.g., [35,36], and there is also evidence that a positive treatment response is linked to the number of semantic characteristics PWA generate during treatment [37]. 

#### 2.2.3. Verb Network Strengthening Treatment 

Verb Network Strengthening Treatment (VNeST) also aims to facilitate lexical retrieval and is based on the theoretical model that highlights the central role of verbs in semantics and syntax (see [38]). During treatment, PWA are prompted to generate thematic roles (i.e., agent and patient) for trained verbs. A single verb can generate many thematic role pairs that all reinforce the target verb. In addition, PWA are asked WH-questions (e.g., why, where, when) about explicit thematic roles for each trained verb. This way, treatment gradually targets the facilitation of sentence production and discourse. Several studies support the potential of VNeST to aid the lexical retrieval of single words, sentences, and discourse in PWA with different types and severities of language deficits e.g., [39,40]. However, collectively, the number of participants investigated in this type of treatment remains low and further research is needed to support VNeST efficacy and effectiveness. 

#### 2.2.4. Sound Production Treatment 

Sound Production Treatment (SPT) is a type of articulatory–kinematic treatment [41] whereby incorrectly produced sounds (i.e., monosyllabic and polysyllabic words, phrases, sentences) are practiced hierarchically through modeling, repetition, minimal pair contrast, orthographic cuing, integral stimulation (i.e., “watch me, listen to me, say it with me”), and articulatory placement instructions. This treatment is not a therapeutic approach to aphasia per se, but more so for apraxia of speech (AoS). However, AoS appears alongside post-stroke aphasia subtypes as well as several aphasia etiologies (e.g., non-fluent progressive aphasia), and, for that reason, SPT is widely used in aphasia rehabilitation. There is evidence that SPT leads to language gains in trained and untrained word production, phrases, and sentences [41]. 

#### 2.2.5. Treatment of Underlying Forms 

Treatment of Underlying Forms (TUF) is based on generative syntax [42] and targets deficits exhibited at the sentence level in people with agrammatic aphasia by training the production of grammatically complex sentences [43]. PWA are provided with written cards that have the components of simple active declarative sentences (e.g., subject, verb) and a picture illustrating the action. After identifying the verb, PWA are trained to reorder sentence components to produce more complex sentences. There is evidence supporting TUF-related improvements in complex sentence production [44] and generalization for untrained and less complex sentences [45]. 

#### 2.2.6. Constraint-Induced Language Therapy 

Constraint-Induced Language Therapy (CILT) is a treatment approach for expressive language difficulties. The key component of CILT is the forced use of spoken language and the restraint of all other communication modalities (e.g., hand gesturing). Shaping (i.e., modification of linguistic requirements) is another feature of CILT which requires more difficult language goals as treatment progresses. CILT is known for its intensive approach necessitating massed practice [46]. The central activity of CILT is the “Go Fish” game in which a person asks another for a card that matches one of their own. If the other individual possesses the requested card, it is given to the requestor. If not, then the requestor must “go fish” (i.e., draw a card from the deck). When one of the players no longer holds any unmatched cards, the game is over. Pulvermüller et al. [47] conducted the first CILT study with favorable results. Several studies support CILT-related language gains in PWA e.g., [48,49]. More recently, it was found that CILT delivered in both intensive and distributed dosages had beneficial effects on both standardized and discourse measures [50]. 

#### 2.2.7. Melodic Intonation Therapy 

Melodic Intonation Therapy (MIT) was first developed to recruit right hemispheric brain regions related to the awareness of melody and rhythm to improve expressive language in individuals with non-fluent aphasia [51]. The two main components of MIT are (i) rhythmic tapping of the left hand that accompanies the production of syllables, and (ii) exaggeration of the natural prosody of speech [52]. There is emerging evidence favoring the efficacy of MIT [53,54], and, for that reason, several modified MIT protocols have been evaluated by different research centers and clinical settings e.g., [55]. 

### 2.3. Additional Therapy Approaches

There are several other therapeutic approaches that are used in aphasia rehabilitation. For example, “Response Elaboration Training (RET)” is a treatment strategy that aims to improve the informational load and length of an individual’s utterances, focusing on spontaneous responses to action pictures [56]. “Promoting Aphasics’ Communicative Effectiveness (PACE)” takes advantage of natural conversation and allows multiple communication strategies (e.g., speaking, writing) [57]. “Oral Reading for Language in Aphasia (ORLA)” aims to enhance reading comprehension in PWA [58].

There is robust evidence supporting the benefits of computerized aphasia therapies [59] derived via computers, smartphones, or tablets without the physical help of a clinician. The use of technology for PWA allows for long-term and low-cost therapy options, and is especially suitable for PWA that live in remote areas and do not have access to SALT services. During the COVID-19 pandemic, technology-supported aphasia therapy was highly valued by PWA and their caregivers, as therapy interruption and fragmentation were prevented. The “EVA PARK” online virtual environment, which simulates a fantasy island context that contains several locations (e.g., houses, coffee shops, a disco) is one software that has been studied [60]. PWA are represented by personalized avatars and communicate with others via speech or written language in various virtual settings. The virtual island offers various scenarios and situations that simulate real-life communication contexts that address specific language goals, promoting active participation and learning. The “EVA PARK” technology is considered accessible, acceptable, and engaging to PWA, and has the potential to drive language gains [61]. In addition, it has been found that by using this virtual platform, PWA feel comfortable and safe and appreciate the opportunity to interact with other PWA [62]. 

Importantly, technology in aphasia rehabilitation is also used in the form of alternative and augmentative communication (AAC) systems to compensate for, temporarily or permanently, the loss of speech. Low-tech AAC systems include the use of objects, pictures, written keywords, and communication books, whereas high-tech ACC systems can include speech-generating devices. Even though there is a variety of ACC systems, many PWA abandon them for several reasons (e.g., cognitive barriers, system complexity) over time [63]. The barriers leading to abandonment need to be evaluated and addressed to make AAC accessible, user-friendly, and acceptable for PWA, their families, and their immediate community. 

Finally, two general therapeutic approaches (i.e., “Communication Partner Training (CPT)” and “Aphasia Communication Groups” (ACGs)) are adopted in aphasia rehabilitation. “Communication Partner Training (CPT)” trains communication partners (e.g., caregivers, friends) on how to best support interaction and communication for PWA [64]. In ACGs, PWA together with their communication partners interact all together on a regular basis. Interestingly, there is increasing evidence supporting the multifaceted benefits of ACGs for PWA (see [65]). For instance, improvements in conversational skills and communication strategies [66]; psychological well-being [67]); psychosocial adjustment [68]; and social connectedness [69] have all been reported.

### 2.4. Non-Invasive Brain-Stimulation-Based Therapies

Overall, even though the evidence indicates that behavioral aphasia rehabilitation leads to significant improvements in communication, the effect sizes of behavioral aphasia treatment studies are somewhat small (=0.28 when comparing SALT with no SALT [22]), and this may lead to only moderate improvements in most cases. This observation has urged aphasiologists to explore complimentary aphasia rehabilitation pathways, such as NIBST. 

Over the last two decades, two NIBST (i.e., Transcranial Magnetic Stimulation (TMS) and transcranial Direct Current Stimulation (tDCS)) have been investigated in post-stroke aphasia research for their potential to enhance neural plasticity and facilitate language recovery. Seizures and other adverse effects are minimal when NIBST are administered within the specified stimulation guidelines [70]. 

To induce language gains in aphasia, both rTMS and tDCS aim to either downregulate neural activity in contralesional brain regions through inhibitory stimulation protocols (i.e., low frequency (LF) rTMS or cathodal tDCS) or upregulate neural activity in perilesional brain areas of the affected hemisphere through excitatory stimulation protocols (i.e., high frequency (HF) rTMS or anodal tDCS). Both approaches are based on two proposed theoretical models of reorganization of language networks post-stroke. The first is the “interhemispheric competition model” [71], according to which there exists a mutual and balanced inhibition between the brain hemispheres. Stroke-induced damage to one hemisphere disrupts this balance leading to reduced inhibition from the affected to the unaffected hemisphere. The unaffected hemisphere, in turn, increases its inhibitory signals to the affected hemisphere. Eventually, activity is decreased in the affected and increased in the unaffected hemisphere. Over the years, it has been reported that the observed activation in unaffected language brain regions of the right hemisphere is deleterious to recovery e.g., [72,73]. Based on this assumption, by downregulating contralesional homologous brain regions via inhibitory stimulation NIBST protocols, language recovery can be induced. The second model suggests that perilesional regions of the left hemisphere are recruited to subserve the reorganization of language networks [74]. This means that, by upregulating perilesional brain regions via excitatory stimulation NIBST protocols, language gains can be achieved. 

Regarding TMS aphasia research, studies have explored the effectiveness of TMS on language gains in all stages of recovery: post-acute e.g., [75,76], subacute e.g., [77,78], and chronic e.g., [79]. Most trials have investigated the effects of LF TMS over the contralesional inferior frontal gyrus (IFG) followed by SALT. The therapeutic potential of LF rTMS has also been reported as a standalone treatment e.g., [80,81,82]. Studies using HF rTMS on perilesional tissue in the left frontal regions [83,84,85] or the left dorsolateral prefrontal cortex (DLPFC) [86] are also promising for language and cognitive gains post-stroke. Regarding the evidence from tDCS studies, cathodal tDCS over the right hemisphere e.g., [87], anodal tDCS over perilesional areas e.g., [88], and simultaneous cathodal and anodal tDCS e.g., [89,90] can induce favorable language effects in PWA. In current NIBST aphasia-related studies, especially tDCS trials, NIBST are used concurrently with behavioral therapy to maximize language gains in PWA. 

Findings suggest that NIBST may drive language improvement in PWA post-stroke. Nevertheless, a recent review of systematic reviews reported that the evidence of LF rTMS for aphasia rehabilitation is inconclusive [91]. In addition, a recent meta-analysis indicated that even though tDCS has the potential to enhance the naming of nouns, it does not appear to improve functional communication in PWA post-stroke [92]. Overall, even though NIBST are promising tools for boosting aphasia recovery, larger-scale, long-term studies are needed (i) to test different protocols tailored to individual needs, (ii) to clarify the precise mechanisms of rTMS and tDCS underlying language recovery, and (iii) to determine which PWA are good responders to those treatment modalities and why others are not. 

To conclude this section, there is no doubt that aphasia therapies need to be relevant and meaningful to every individual with aphasia and his/her family. Interventions should foster a positive impact on QoL and social well-being, enabling the individual to live a fulfilling life with aphasia. 

## 3. Can We Predict Which PWA Will Respond to Treatment?

It could be assumed that as several factors may influence treatment outcomes (e.g., age, type and severity of aphasia, comorbidities, motivation, and engagement), predictions can be made about who responds to aphasia treatment. Nonetheless, predicting aphasia recovery is inherently challenging due to the complex interplay of various factors. 

### 3.1. Individual Variability 

Individual variability plays an important role in therapy response. Age at stroke onset, sex, education, handedness, psychosocial factors, cognitive abilities, and genetics are all factors that may predict treatment outcomes. As the brain’s cognitive processing capacity naturally declines with age, it could be hypothesized that the brain’s plasticity also decreases. Hence, it would be reasonable to expect limited language recovery in older PWA. Indeed, there are studies that have found that people that experience aphasia at a younger age tend to have better outcomes in comparison to people that manifest aphasia at older ages e.g., [93,94]. Nevertheless, the association between age and recovery varies between studies [95,96] and this variation may be attributed to several factors (e.g., comorbidities, differences in sample characteristics and assessment methods). 

With regard to sex, even though it has been reported that women tend to have better language recovery than men post-stroke [97,98], other studies have not demonstrated differences in aphasia recovery that depend on sex e.g., [99,100]. Education is not a reliable predictor of aphasia recovery either, as the relationship between education and language improvement varies across studies [101]. Therefore, further research is needed to determine the underlying mechanisms that contribute to the influence of sex and education on language recovery post-stroke. As for handedness, left-handed children demonstrate more bilateral language representation in comparison to right-handed children, but this difference diminishes in adults [102]. Therefore, based on bilateral representation, it remains unclear whether left-handedness confers an advantage for language recovery [103]. The literature around the association of psychological factors (e.g., social support and mood) and aphasia recovery is limited but indicates their possible impact on the well-being and independence of PWA [104]. With regard to cognitive skills, a recent study found that PWA post-stroke with better attention and working memory functions at baseline had better language outcomes post-SALT [105]. Other studies have also found similar results e.g., [106,107], suggesting that cognitive functions may have an impact on aphasia treatment response. 

Finally, the influence of genetics on aphasia rehabilitation is an area of ongoing research. The brain-derived neurotrophic factor (BDNF) plays an important role in learning and memory by inducing long term potentiation (LTP) in neural cells [108]. A recent study demonstrated that BDNF polymorphism can influence aphasia recovery. Particularly, it was found that the *Val*66*Met* polymorphism of BDNF is associated with poorer language recovery [109]. However, the participants of that study differed in aphasia chronicity. In another study [110], no association between BDNF genotype and language performance was observed. But, in that study, important factors, such as lesion size and stroke severity, were not taken into consideration. The existing evidence on the effects of BDNF polymorphism on aphasia recovery is limited and inconsistent. The conflicting results and methodological limitations highlight the need for further research to better understand the role of BDNF polymorphism in aphasia rehabilitation outcomes [111]. 

To gain a clear understanding of how individual variability can influence aphasia recovery, future research should include individuals with diverse aphasia profiles, utilize standardized assessment measures consistently, and try to address all possible confounding factors. 

### 3.2. Cortical Integrity 

#### 3.2.1. Aphasia Severity, Location of Brain Injury, and Lesion Size 

Aphasia classification is intricately linked to lesion location and lesion size and correlates with aphasia severity, but when it comes to predicting treatment response, the impact of aphasia type remains uncertain [93]. For some PWA, aphasia severity may predict short- [112] and long-term outcomes [113]. There is robust evidence supporting that higher severity is associated with lower rates of spontaneous recovery and poorer response to treatment [114,115]. Recently, it was found that PWA with mild language impairments demonstrated positive responses to semantically focused therapy, while those with more severe symptoms benefited from phonologically focused treatment [116]. Thus, it seems that aphasia severity not only has an impact on overall language recovery but also influences the response to different treatment modalities [93]. 

According to the network organization of language in the brain, communication hubs/networks play a critical role in language processes, and damage to those networks has a greater negative impact on language impairments and recovery in comparison to lesions in non-hub regions [93]. For instance, there is robust evidence that links adverse recovery outcomes to lesions in the temporoparietal junction (including posterior temporal and inferior parietal areas) e.g., [117,118]. Nonetheless, other findings have revealed that PWA with greater damage in anterior areas but with intact basal ganglia responded better to aphasia therapy compared to PWA with larger lesions in posterior brain regions [119]. Even though it is reasonable to assume that larger lesions may affect language network hubs and consequently confer a greater negative impact on language recovery, findings regarding lesion magnitude are controversial. While it has been shown that larger lesions are associated with poorer recovery e.g., [120], there is evidence that this is not always the case [121]. Considering the existing evidence connecting the location and size of lesions, it is important to understand their combined impact on aphasia recovery. 

#### 3.2.2. Neural Plasticity 

Even though the course of recovery is different for every person with post-stroke aphasia, particular stages in the recovery process are common for all afflicted individuals. Stroke triggers several molecular cascades that promote spontaneous neural repair [122]. The mechanisms of spontaneous recovery are strong for several weeks after the stroke [4] and start to decline in the chronic phase, which by convention is considered as more than six months post-stroke [123]. Overall, several brain-mapping stroke studies report that language-related brain reorganization is a dynamic process showing activation shifts over time. Specifically, in the early stages, cortical activity is initially reduced at the site of the lesion and as time passes it Increases again [124]. This suggests that the left hemisphere is better equipped to support language abilities [125]. Despite the proposition that the observed right hemispheric activation is a passive event reflecting reduced interhemispheric inhibition [126], there is strong evidence supporting that activity in residual, unaffected contralesional hemispheric areas may also contribute to functional recovery for lost functions supported by damaged areas [71,127,128,129].

#### 3.2.3. Speech and Language Therapy (SALT) 

In 2006, a comprehensive review of the relationship between time post-onset of aphasia and response to treatment in chronic patients revealed that response to SALT is not linked to time post aphasia onset [130] and this finding was confirmed a decade later [131]. However, recent aphasia research supports that initiating aphasia therapy in the early phase post-stroke (i.e., <1 month post-stroke) leads to better outcomes compared to commencing treatment in the chronic phase (i.e., >6 months post-stroke) [97]. And, even though clinicians tend to favor early post-onset commencement of aphasia therapies, recent reports suggest that there are no convincing neurobiological reasons for restricting aphasia therapy to the first few months after the stroke [132]. Overall, it is believed that specific aphasia interventions can modify the brain’s structure and function [133] and this may work in favor of aphasia recovery. For example, there is evidence that melodic intonation therapy leads to structural changes in the arcuate fasciculus (AF) of the right hemisphere [134], and anomia therapy causes structural changes to the left AF [135]. As for functional changes, it has been shown that SFA may lead to functional connectivity changes in the default language network (DLN) that drives language gains [136]. 

Two key treatment components (i.e., treatment dosage and intensity, where intensity equals frequency and duration) are crucial factors that affect aphasia treatment outcomes. Even though it has been found that higher intensities and doses of SALT yield greater treatment effects [22], lower doses of therapy, such as multi-modality aphasia therapy (M-MAT) [137], have also proven efficacious. Recently, it was reported that more than two hours of daily SALT within 4 weeks is not beneficial, but even a small increase in treatment duration (i.e., two more weeks of treatment) leads to substantially better results [138]. In the recent systematic review of Husak and colleagues [24], the effects of SALT initiated within four months of stroke-induced aphasia onset were explored; it was reported that none of the reviewed studies found that increasing the weekly amount of therapy resulted in improvements in primary outcome measures. Another recent systematic review [139] verified that the measurement and reporting of SALT dose across post-stroke aphasia treatment trials is neither consistent nor systematic, and even though there is evidence of a relationship between treatment dose and efficacy, the variability and combinations of SALT treatment schedules (i.e., session dose and frequency and duration of therapy) may mask the dose and efficacy relationship [139]. 

In terms of intensity, intensive programs are based on the idea that long-term neuroplastic changes associated with recovery are induced by intensive practice [140]. One study [141] reported that compared to massed practice distributed over three weeks, distributed therapy over eight weeks yielded significantly better results in naming abilities. However, this was not the case for functional communication and communication-related QoL immediately after treatment and at one-month follow-up. Even though motor learning and cognitive trials provide evidence that distributed practice may be more beneficial [142], it is still unclear whether massed practice is superior to distributed SALT in aphasia rehabilitation [143]. 

To conclude, reliable approaches for predicting aphasia treatment response remain evasive due to the complex and heterogeneous nature of this acquired condition. Integrating multimodal approaches that combine various data sources (e.g., biomarkers, behavioral and neuroimaging measures) holds promise in refining predictive models of aphasia recovery and tailoring interventions to individual needs, ultimately optimizing aphasia recovery.

## 4. Challenges in Participant Recruitment 

Aphasia rehabilitation research informs our understanding of brain functions and language recovery post-stroke, contributes to our diagnostic reasoning, motivates treatment recommendations, and, overall, shapes clinical aphasia practice. Recruiting participants for aphasia studies becomes a multifaceted endeavor that requires innovative strategies, enhanced sensitivity, and collaboration among researchers, clinicians, and support networks. An overview of challenges faced by researchers in recruiting PWA for aphasia rehabilitation studies is explored here. This serves as a reflective exercise with the aspiration that researchers will further contemplate ways to improve participant recruitment in aphasia rehabilitation studies. For ease of reference, the identified challenges have been categorized into three types:

(1) The nature of the aphasia post-stroke;
(2) Study size including participant and carer-related barriers;
(3) Regulatory barriers and clinical priorities.

### 4.1. Nature of the Aphasia Post-Stroke 

Aphasia is a highly heterogeneous condition with variations in its underlying cause, symptom profile, and functional limitations. It is difficult for researchers wanting to explore the effects of a specific therapeutic program on specific language deficits to be able to recruit, at the appropriate time, an adequate sample of PWA (i) at the same stage of aphasia recovery, (ii) presenting with the same aphasia type and severity, and (iii) exhibiting the same language deficits. In addition, communication deficits per se (especially severe) in the acute, subacute, or chronic phase with or without other concomitant health issues (e.g., cognitive problems, agitation, confusion, physical and/or emotional fatigue) undermine accurate language assessments and full participation (i.e., recruitment and retention) in research trials. Inaccurate assessments and incomplete research sessions are sound reasons for exclusion from trials. 

In aphasia research, addressing the heterogeneity within the aphasia population is of paramount importance, and, for this to be achieved, well-designed inclusion and exclusion criteria must be implemented. Such criteria can allow researchers to capture the diverse characteristics and needs of PWA, enabling them to tailor interventions accordingly. The careful selection of participants, according to specific inclusion and exclusion criteria, can create a balance between homogeneity and diversity. Homogeneity in terms of aphasia traits allows the evaluation of treatment efficacy and effectiveness, and diversity is essential for the generalizability of research findings to the broader population of PWA.

### 4.2. Study Size

To ensure the establishment of evidence-based aphasia therapies, patient participation in aphasia trials is of paramount importance. Unfortunately, a key limitation of aphasia rehabilitation studies concerns small sample sizes. In comparison to other conditions, the prevalence of aphasia is relatively low (up to ≈ 30% in chronic aphasia, [144]) and such rates result in smaller pools of possible participants. This scarcity makes it challenging to recruit PWA that meet specific inclusion criteria in aphasia trials. A small sample size reduces (i) the scientific rigor of the study results, (ii) the possibilities that the sample adequately represents the target population, and (iii) the chances that findings can be generalized to other PWA. In addition, when recruitment is too low, clinical trials are canceled, and new, promising therapies are abandoned [145]. To overcome challenges in recruiting PWA for aphasia rehabilitation trials, researchers should, indicatively, (i) foster strong collaborations with clinicians from several disciplines and support networks across multiple institutions nationally and internationally, (ii) adopt innovative approaches, and (iii) ensure participant-centric study designs [146]. 

#### 4.2.1. Participant-Related Barriers

With regard to participant barriers, as aphasia affects language abilities, it is often very difficult for PWA to effectively express their interest in participating in aphasia trials. Finding alternative communication methods (e.g., AAC systems) becomes essential in bridging this gap. Furthermore, PWA usually have concomitant cognitive impairments (problems with attention and memory, etc.) that negatively affect their ability to engage in study procedures (e.g., remember appointments, comprehend study procedures, etc.). As a result, researchers need to adapt their recruitment strategies in such a way that facilitates participation, for example, by providing aphasia-friendly study materials and offering repeated reminders. Moreover, due to aphasia’s impact on information processing and social engagement, PWA may have a limited awareness of available research opportunities. Currently, a framework for conducting and reporting the involvement of PWA in qualitative participatory research studies is lacking and this limits effectiveness to promote equitable best practice in aphasia rehabilitation [146]. Therefore, it is suggested that researchers should collaborate with other clinicians from the same or different disciplines, aphasia support networks, and advocacy organizations to (i) raise aphasia awareness, and (ii) actively reach out to potential participants to educate them and their carers as well. Furthermore, as aphasia can have adverse emotional and psychological effects for PWA, willingness to engage in research even for therapeutic purposes may decrease. Researchers must be sensitive to the emotional needs of PWA, establish rapport, and provide appropriate emotional support throughout the recruitment and retention process.

#### 4.2.2. Carer-Related Barriers

Carers very often have multiple life responsibilities, making it challenging to have time available for participation in research studies. This is especially true when research designs are complex, with extensive selection criteria and requiring several lengthy baseline and follow-up assessments together with lengthy therapeutic appointments. The obstacles for recruitment escalate when potential participants have physical problems and accessibility issues (e.g., large geographical distance from study sites, transportation difficulties). To avoid selection bias and ensure recruitment and retainment, researchers can explore options for remote participation (e.g., online surveys), telehealth appointments, and home-based therapies. Also, the provision of financial assistance with transportation or arrangement for local study sites can also enhance accessibility and encourage study engagement. By offering flexible scheduling options (e.g., evening or weekend appointments) and by minimizing the duration and frequency of study visits, a reduction in the time burden on caregivers is achieved.

Additionally, carers may be discouraged from taking part in a trial by the potential costs associated with engagement. To alleviate financial barriers, financial support for transportation purposes or offering financial compensation for study retention may incentivize caregivers to engage in research trials. Furthermore, recruiting participants for aphasia rehabilitation studies can be challenging due to the emotional and physical strain experienced by caregivers, making it difficult for them to take on additional commitments, such as study participation. Such emotional barriers can add significant challenges to recruiting participants in aphasia trials. To alleviate the emotional and physical strain on caregivers, researchers can implement support mechanisms, such as counseling services or in-home respite care, to assist with the activities of daily living and provide companionship if needed. Such strategies can create a more supportive environment for caregivers, alleviate the stress they experience, and enhance their involvement in aphasia studies. As previously reported in a study by Charalambous and colleagues [146], caregivers may worry that the participation of their loved one in trials could be, for the individual with aphasia, frustrating, overwhelming, tiring, or inefficient. To deal with this issue, researchers can adopt an inclusive participatory approach by considering caregivers as active participants. In particular, caregivers could be involved in assessment processes and treatment goal setting. By recognizing and valuing the crucial role of caregivers in assessing and treatment planning, researchers may foster their motivation and active involvement in the study. Other obstacles for study engagement on behalf of carers could be the limited awareness or understanding of research opportunities, or the potential benefits of participation. Collaboration between researchers and healthcare professionals and support organizations could help with the effective dissemination of information about the purposes, goals, and favorable impacts of aphasia trials on PWA and society. 

### 4.3. Regulatory Safeguards and Clinical Priorities 

Further considerable obstacles faced by researchers in recruiting PWA for aphasia rehabilitation trials are associated with research regulatory barriers and clinical priorities. These challenges arise from the complex interplay between study protocols, regulatory requirements, and the prioritization of clinical interventions. Regulatory safeguards can appear in the form of rigorous ethical guidelines, institutional review board (IRB) approvals, and legal issues that researchers have to consider when conducting human-related trials. Complying with the necessary regulatory frameworks usually necessitates extensive paperwork and close monitoring to ensure adherence to guidelines. On the one hand, such regulations are important as they guarantee participant safety and ethical conduct, but, on the other hand, they may cause delays in the recruitment process and present additional administrative burdens to researchers.

Due to the prioritization of patient care in healthcare settings, the focus of healthcare services is typically on clinical responsibilities. Consequently, healthcare professionals may have limited time and resources to contribute to research activities. Indeed, there is evidence indicating that clinicians may perceive research activities as an obstacle to providing patient care in stroke units, and this could lead to their resentment towards the physical presence of researchers [147]. Balancing the need for rigorous research protocols and regulatory compliance with the clinical priorities of delivering immediate and direct care to PWA can hinder recruitment efforts. There are various approaches to surmount this challenge, including integrating research activities within existing clinical workflows, streamlining research processes, and emphasizing the possible advantages of research participation for both the PWA and the overall field of aphasia rehabilitation. In addition, it is very important to establish efficient communication channels among researchers, administrators, clinical stakeholders, and regulatory bodies. This would provide reassurance that the research complies with regulatory requirements, and would address any concerns, minimizing unnecessary burdens on clinical practice. 

## 5. Conclusions and Recommendations 

Numerous therapeutic protocols and complimentary rehabilitation strategies are available, either used in isolation or combined with other therapies to effectively rehabilitate aphasia. The integration of multimodal approaches that combine various data sources (e.g., biomarkers, behavioral and neuroimaging measures) holds promise in refining predictive models of aphasia recovery and tailoring interventions to individual needs, ultimately optimizing aphasia rehabilitation. The pursuit of further development in the efficacy and effectiveness of aphasia treatment strategies and a deeper understanding of aphasia recovery processes necessitate the successful recruitment of more PWA to research studies. For this to be achieved, a research culture embracing PWA, their carers, and clinicians needs to be fostered and sustained globally, especially in allowing PWA to prioritize their own research needs. 

## Data Availability

Data is contained within the article.
